# How Do Air Quality Issues Caused by Particulate Matter Affect Consumers’ Emotional Response to Tourism Destinations and Willingness to Visit?

**DOI:** 10.3390/ijerph181910364

**Published:** 2021-10-01

**Authors:** Jongsik Yu, Kyeongheum Lee, Antonio Ariza-Montes, Alejandro Vega-Muñoz, Heesup Han

**Affiliations:** 1 College of Business Division of Tourism and Hotel Management, Cheongju University, 298 Daesung-ro, Cheongwon-gu, Cheongju-si 28503, Korea; andyjs.yu@gmail.com; 2 College of Hospitality and Tourism Management, Sejong University, 98 Gunja-dong, Gwanjin-gu, Seoul 143747, Korea; heum112311@gmail.com; 3 Social Matters Research Group, Universidad Loyola Andalucía, C/Escritor Castilla Aguayo 4, 14004 Córdoba, Spain; ariza@uloyola.es; 4 Public Policy Observatory, Universidad Autónoma de Chile, Santiago 7500912, Chile; alejandro.vega@uautonoma.cl

**Keywords:** perceived risk, trust, desire, customer return on investment (CROI), willingness to visit a tourism destination, alternative attractiveness

## Abstract

This study identifies the perceived risk factors of particulate matter (PM) and the effect of the perceived risk factors of PM on the relationship between tourists’ trust and aspiration regarding the tourist destination, the customer return on investment, and the willingness to visit a tourism destination. Accordingly, this study discussed the severity of PM, which plays a key role in causing air quality issues, and classified the factors for perceived risk of PM into physical, psychological, financial, functional, and time risks to verify its effect on consumers’ emotional response and willingness to visit. Data collection for empirical analysis took place in April 2021 for two weeks. A total of 285 significant data points were obtained on tourists with travel experience in the past year. The demographic characteristics were confirmed using SPSS 22.0 (IBM, New York, NY, USA) and AMOS 22.0 (IBM, New York, NY, USA), and the measurement and structural models were verified through a confirmatory factor analysis and structural equation modeling, respectively. The empirical analysis showed that the perceived risk of PM has a negative effect on trust in the tourism destination and desire for it, and the behavioral intention of customers. Furthermore, alternative attractiveness was found to play a significant moderating role. The results of this study proved the negative effect of PMs on tourism destinations and provided implications and insights to present a meaningful strategy for minimizing PMs’ perceived risk.

## 1. Introduction

Increased concentrations of particulate matter (PM) and air pollution have detrimental effects on human health and are becoming a serious problem worldwide as they are known to be the leading cause of human death and diseases [1]. In general, PM can be divided into two types according to their size. It can be divided into PM10, which is fine dust with a particle diameter of less than 10 μm, and PM2.5, which has a particle diameter of less than 2.5 μm [2]. PM can be very detrimental for human health. Specifically, inhaling PM10 can irritate human eyes, nose, and throat, and ultrafine particles such as PM2.5 particles can penetrate human lungs and blood, causing very serious diseases [3]. Moreover, PM causes many different diseases, such as stroke, heart disease, lung disease, asthma, and acute respiratory disease [4]. Therefore, the WHO considered the negative effects of PM on humans and designated it as a group 1 carcinogen (Group 1) [5].

Particulate matter (PM) is one of the factors that can reduce the quality of life of the Korean people and checking the PM level has become part of their daily routine. Checking the PM level on a daily basis has become essential for the public. According to Greenpeace [6], South Korea has the most severe PM2.5 pollution among OECD member countries, and 61 Korean cities were included in the list of the top 100 cities in OECD member countries with the most serious PM2.5 pollution. This PM problem can develop into a factor that hinders outdoor activities, and further, it can lead to a recession in the tourism market and reduce the attractiveness of a tourism destination. The number of inbound tourists visiting South Korea in 2019 was 17.5 million, which was an all-time high, and it aided the revitalization of the domestic economy by generating a production inducement effect of USD 46 billion [7]. However, PM is highly likely to have adverse effects such as a decrease in the number of tourists and a decrease in tourism revenue. Accordingly, for the development of the Korean tourism industry, there is a need to maintain and develop Korea’s attractiveness as a tourism destination, and to minimize factors hindering the development of the tourism industry. 

Tourists perceive PM as a potential health risk or a factor limiting travel, and they are very likely to change or cancel travel plans because of it [8,9]. Hence, PM is a negative factor that can reduce the number of tourists and reduce the desire to visit. As such, PM in the tourism industry is a threat that causes many problems, and hence, many studies have studied the issue of PM. However, an examination of existing studies on PM reveals that there are very few that investigate the effect of PM on the tourism industry. Accordingly, the main purpose of this study is to understand the perceived risk factors for PM and the impact of perceived risk of PM on the domestic tourism market. Specifically, the study examines 1) the perceived risk of PM, 2) the effect of the perceived risk of PM on trust in the tourism destination and desire for it, 3) the effect of trust in and desire for tourism destinations on customer return investment, and 4) the impact of customer return investment on the willingness to visit the tourism destination. Finally, this study investigates the moderating effect of alternative attractiveness on the relationship between the perceived risk for PMs and trust in and desire for tourism destinations.

## 2. Literature Review

### 2.1. Perceived Risk

Perceived risk in consumer behavior has been regarded as a key factor influencing customer behavior and intention [10,11]. Perceived risk can be defined as a consumer’s subjective belief in the risk involved in achieving the desired outcome [12]. In other words, there is a certain level of risk because consumers cannot easily predict the risks that may arise in the process of choosing certain products and services. Therefore, perceived risk can be seen as a subjective feeling of uncertainty about the outcome from the viewpoint of consumer behavior (e.g., loss or failure) [13]. Perceived risk in consumer behavior can be divided into several components according to the nature of risk perceived by consumers [11,14]. According to Jacoby & Kaplan [15], perceived risks can be divided into physical, psychological, functional, social, and financial risks. McCorkle [16] expanded the study of perceived risk to include time risk [17]. The previous studies on perceived risk have the commonality that most of them consider physical, psychological, financial, functional, and time risks [10,11,18]. 

Since different types of perceived risk may cause resistance in consumers’ decision-making process, perceived risk plays a critical role in explaining how consumers make decisions [11]. In other words, perceived risk influences consumer’s decision-making and behavior according to the level of uncertainty perceived by consumers [19,20]. As such, perceived risk has a direct impact on consumer behavior and intention, and hence, many studies have verified the relationship between perceived risk and consumer behavior and intention. Specifically, Jung & Han [21] claimed that perceived risk has a significant effect on the purchase intention of travelers and negatively affects the emotions, motives, and behavioral intention of travelers. Moreover, Han et al. [10] found that perceived risk of electric planes has a negative effect on attitude and the formation of trust for electric planes. According to previous studies, air pollution does affect the tourist decision-making process. Lapko et al. [22] investigated how air pollution affects people’s travel plans and found that air pollution affects the duration of travel, the travel motivation (e.g., business trip or leisure travel), and the decision of taking children along or not. Hence, in this study, the following hypotheses were established based on previous studies.

**Hypothesis** **1** **(H1).** *Perceived risk of PM will reduce trust in tourism destinations.*

**Hypothesis** **2** **(H2).** *Perceived risk of PM will reduce desire for tourism destinations.*

### 2.2. Trust in and Desire for Tourism Destinations

Trust can be considered an essential component influencing customer decision-making and purchasing behavior, and it is sometimes described as an essential determinant [23]. Customers often choose products and services they can trust because purchasing such products and services reduces the risk of uncertainty of functionality and quality [10,19]. The certainty of this exchange relationship plays a very important role in improving the orientation of the relationship between customers and the service provider, which enables them to engage in relationships and reduces risk perception [18]. As such, the importance and necessity of trust has been proven in many studies. In particular, trust was found to play a very important role in the field of tourism consumer behavior. Specifically, according to Chaudhura & Holbrook [24], the trust formed in the mutual exchange relationship has a positive effect on the formation of consumer commitment, attitude, and loyalty. Han & Hyun [25] claimed that high levels of trust that travelers have in the service provider is associated with more positive behavior and intention toward the service provider.

In a business context, desire can be defined as an intangible psychological component that describes a consumer’s individual passion to use or purchase products and services [26,27]. In addition, desire is a state of mind in which an individual has a personal motivation to perform a specific action or achieve a goal [27,28]. Desire can appear as a positive or negative response through direct/indirect influence, which can lead to intention formation and action. For instance, Perugini and Bagozzi [27] explained that desire is a powerful factor triggering the intention for a specific behavior. According to Han et al. [29], desire exerts direct/indirect effects through an important mediating role between cognitive/emotional factors and outcome variables, such as intention and behavior, and increases the strength of emotional bonding through loyalty intention, for example. Therefore, in this study, the following hypotheses were established based on previous studies.

**Hypothesis** **3** **(H3).** *Trust in the tourism destination will have a positive effect on customer return on investment.*

**Hypothesis** **4** **(H4).** *Desire for the tourism destination will have a positive impact on customer return on investment.*

### 2.3. Customer Return on Investment (CROI) and Willingness to Visit a Tourism Destination

Customer return on investment (CROI) is related to the value perceived during the consumption experience and is closely related to the practical aspects of the consumption experience traded in return for consumption [30]. Furthermore, customer return on investment consists of an active investment in potentially profitable financial, time, behavioral, and psychological resources [31]. Given the characteristics of the customer return on investment, it is a reward for the resources (e.g., money, time, and effort) invested in the consumer experience, and consumers expect higher rewards with greater investment of resources. According to Mathwicka et al. [31], customer return on investment can be divided into two aspects, which consist of economic utility and efficiency of an exchange encounter. The relationship between customer return on investment and positive consumer behavior can be confirmed from previous studies. Specifically, Kim et al. [32] argued that consumer satisfaction and trust are formed when price fairness versus service quality is appropriate. Ahn et al. [33] claimed that unreasonable financial values perceived by consumers cause negative consumer attitude and behavior. Therefore, customer return on investment can be considered a key factor inducing positive consumer behavior. Accordingly, in this study, the following hypothesis was established based on previous studies.

The importance of retaining and returning customers is further emphasized in today’s highly competitive environment. In particular, increasing the intention to purchase a company’s products and services or visit a tourism destination is very effective because it can save time and money when creating new customers [34]. Thus, retaining and motivating customers to return are critical to a company’s survival and long-term success [35,36]. The willingness to revisit a tourism destination can be defined as the preparation or intention to visit the same destination repeatedly, and it provides the most accurate forecast for revisit [37]. The intention to revisit a tourism destination is the result of satisfaction with the tourism destination [38]. Therefore, elements such as various pleasures, memories, and positive experiences in the tourism destination are essential for revisit.

**Hypothesis** **5** **(H5).** *Customer return on investment will have a positive effect on willingness to visit a tourism destination.*

### 2.4. The Moderating Role of Alternative Attractiveness

Customers often compare the differences in attractiveness between their existing supplier and suppliers of new products and services. If they find the new supplier more attractive, they end the relationship with the old supplier and start a relationship with the new one [39]. Such a result could be reversed. Customers are more likely to maintain their relationship with their existing supplier when they feel that the perceived alternative attractiveness is insufficient [40]. In other words, alternative attractiveness is inversely related to maintaining a positive customer relationship with the existing supplier. In the current situation, wherein the products and services of the tourism industry are diversifying and there are more options to choose from, customers tend to hesitate or get confused about their choices [41]. According to the theory of choice, customers choose products and services with the highest value through alternative-based information processing [42]. Many studies on alternative attractiveness found that alternative attractiveness plays a very important role in explaining an individual’s decision-making process or behavior [43,44,45]. For example, Goode and Harris [43] explained that when customers who have a strong alternative attraction to a particular product or service become aware of the advantages of the alternative attraction, they are more likely to complain about the existing product or service. Moreover, Nagengast et al. [45] argued that customer decision-making or the relationship between post-purchase intention and behavior is significantly influenced by alternative attractiveness. According to Han et al. [44], the intention to act can induce actual behavior, and in the relationship between these two variables, alternative attractiveness plays a role in moderating the actual behavior. Therefore, this study established the following hypotheses to achieve its research purpose based on previous studies.

**Hypothesis** **6a** **(H6a).** *There will be a significant moderating effect of alternative attraction on the relationship between perceived risk of PM and trust in the tourism destination.*

**Hypothesis** **6b** **(H6b).** *There will be a significant moderating effect of alternative attraction on the relationship between perceived risk of PM and desire for the tourism destination.*

### 2.5. Research Model

**Figure 1:**
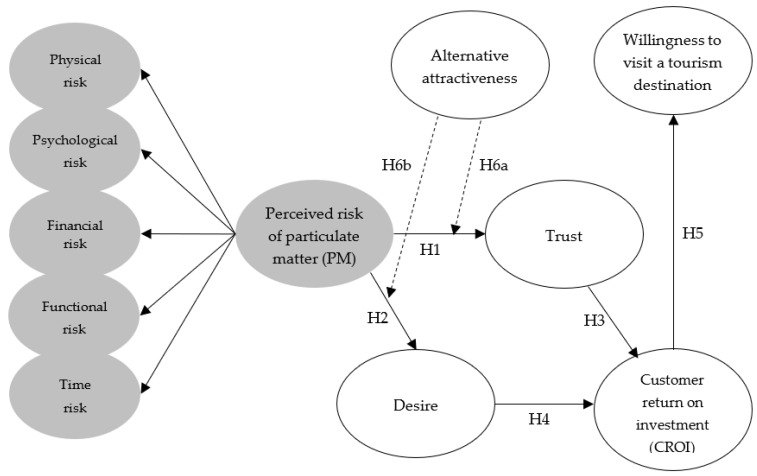
Research model.

Under the theoretical framework, the effect of perceived risk of particulate matter on trust, aspiration, customer return on investment, and willingness to visit a tourism destination is verified. Additionally, the moderating role of alternative attractiveness is verified. A total of seven hypotheses are presented to determine the relationship between the variables presented, and Figure 1 is presented for a clearer understanding. 

## 3. Methods

### 3.1. Measurement Instruments

The questionnaire in this study largely consists of three sections. Specifically, it consists of a description of the study, questions about the variables, and the demographic characteristics of respondents. The measurement items used in this study were those whose validity and reliability have been proven in previous studies. They were modified and supplemented to fit the purpose of this study. A 7-point Likert scale (1= “strongly disagree”; 7= “strongly agree”) is used in this study to measure the answers. Looking at the items in detail, 15 questions were used based on the Dayour et al. [17] and Quintal et al. [11] studies on perceived risk. For trust in the tourism destination, three questions were used based on Morgan and Hunt [46] and Ok and Back [47]. Regarding desire for the tourism destination, three questions were used based on Han et al. [48] and Perugini and Bagozzi [27]. Moreover, three questions were used based on the Mathwick et al. [31] study on customer return on investment. Regarding the willingness to travel to a tourism destination, three questions were used based on Grewal et al. [49]. The first questionnaire of this study was pre-tested with a group of seven experts consisting of three university professors and four tourism practitioners specializing in the field of tourism. Through this, the questionnaire was revised and supplemented to enable respondents to understand the contents of the questionnaire more clearly and accurately.

### 3.2. Data Collection and Sample Characteristics

A quantitative statistical analysis was used to ensure objectivity of the research results. Additionally, an approach was used to test research hypotheses that could be measured quantitatively. Furthermore, the most commonly used survey method was adopted when obtaining meaningful data for use in empirical analysis. The data used in the empirical analysis were collected through the web-based platform of a research firm specializing in online surveys and data collection. The Internet research firm used in this study is located in Korea and has numerous research panels. Respondents were randomly selected using the database of the Internet research institute. Respondents who participated in the survey were assured of the confidentiality of their personal information, and were assured that the responses to the questionnaire would not be used for any purpose other than the study. In addition, for the purpose of research and the collection of clearer and more grounded data, the respondents were limited to tourists who have traveled to Korea at least once in the past year. In total, 291 samples were obtained through this method, and among them, six samples which were determined to have not been answered faithfully, were excluded. Thus, 285 samples were used for empirical analysis. These 285 responses exceeded the minimum number of sample size (n = 110), according to the rules of thumb in sample size estimation. The sample size is sufficient enough to carry out the measurement model testing and the structural model analysis. Regarding the characteristics of the respondents, all were Koreans. In terms of gender, 144 were men (50.5%) and 141 women (49.5%). In terms of the age of the respondents, 98 people were in their 20s (34.4%), 88 people were in their 30s (30.9%), 75 people were in their 40s (26.3), and 24 people were in their 50s (8.4%). Regarding educational level, 23 respondents (8.1%) were college graduates, 233 (81.7%) were university graduates, and 29 (10.2%) were graduates of graduate school or higher. Lastly, in terms of income, 39 people were earning less than $30,000 USD per year (13.7%), 135 people had an annual income between $30,000 and $50,000 USD (47.4%), 78 people had an annual income between $50,000 and $70,000 USD (27.4%), and 33 people had an annual income of more than $70,000 USD (11.5%).

## 4. Results

### 4.1. Confirmatory Factor Analysis

The most useful analysis method used to verify scale validity and reliability is confirmatory factor analysis [50]. Hence, confirmatory factor analysis was performed using the maximum likelihood estimation method to verify the validity and reliability of the scale used in this study. Detailed analysis results are as follows. First, the fit of the measurement model was χ^2^ = 830.677, df = 360, *p* < 0.001, χ^2^/df = 2.307, RMSEA = 0.068, CFI = 0.941, and TLI = 0.928, which is statistically acceptable. In other words, when compared to previous studies on tourism areas, the fitness of the measurement model suggested in this study is appropriate. Next, the reliability of the items measured in this study was verified with standardized regression weights. The reliability of the measurement item is secured when the standardized regression weight is more than 0.5. In the results of this study, all measurement items ranged from 0.637 to 0.927. Accordingly, reliability of all presented measurement items was secured. Next, to confirm convergent validity and internal consistency, average variance extracted (AVE) and composite reliability (CR) were verified. When the AVE value is more than 0.5 and the CR value is more than 0.7, there is no problem with the internal consistency and convergence validity of the measured variable [51]. The AVE values ranged from 0.596 to 0.719, and the CR values ranged from 0.813 to 0.885. Therefore, there is no problem with the internal consistency and convergence validity of the measurement variables presented in this study. Lastly, a discriminant validity test confirmed the differentiation between constructs. Discriminant validity can be examined by the size of the correlation coefficients between the AVE value and the latent variable. If the AVE value is greater than the square of the correlation coefficient, there is no problem with the discriminant validity [51]. The analysis showed that the AVE value was larger than the square value of the correlation coefficient between the variables presented in this study. Accordingly, the discriminant validity among the variables presented in this study was secured. Details are shown in Table 1.

### 4.2. Structural Equation Modeling

In this study, a structural equation was implemented using the maximum likelihood method to verify the hypotheses presented. On the basis of the analysis results (χ^2^ = 838.192, df = 313, *p* < 0.001, χ^2^/df = 2.678, RMSEA = 0.077, CFI = 0.927, and TLI = 0.918), the goodness-of-fit of the structural model presented in this study is considered appropriate. The results of analysis on the second-order factor structure of the perceived risk of PM were examined. The standardized coefficients for the five first-order factors, namely, physical risk, psychological risk, financial risk, functional risk, and time risk were 0.926, 0.923, 0.868, 0.876, and 0.883, respectively. All associations were found to be statistically significant at the *p* < 0.01 level. Further, regarding the R² value, the R² values of physical risk, psychological risk, financial risk, functional risk, and time risk were 0.858, 0.851, 0.753, 0.767, and 0.779, respectively. Each was explained at an appropriate level by the higher-order structure. The model has a higher explanatory power compared to the previous studies’ R² values.

Next, the verification results of the five hypotheses presented in this study were examined. First, the effect of perceived risk of PM on trust in and desire for the tourism destination was verified. The analysis showed that the perceived risk of PM has a statistically significant effect on trust (β = −0.740, *p* < 0.01) and desire (β = −0.761, *p* < 0.01). The results are in line with the research findings of Quintal et al. [11] and Jung & Han [21], which argued that perceived risk inhibits consumer trust and desire. Next, the effect of trust in and desire for the tourism destination on customer return on investment was verified. The analysis showed that both trust in (β = 0.196, *p* < 0.01) and desire (β = 0.774, *p* < 0.01) for the tourism destination have a statistically significant effect on customer return on investment. The results are also consistent with the results of Al-Ansi et al. [26] and Han et al. [10] who insisted that consumer trust and desire produce positive consumer behavior. Lastly, customer return on investment was found to have a statistically significant effect on the intention to travel to a tourism destination (β = 0.587, *p* < 0.01). Accordingly, hypotheses 1, 2, 3, 4, and 5 presented in this study were all supported.

Utilizing a mediating framework within a theoretical model can facilitate the understanding of the complex relationships of the proposed research structure [52]. Therefore, in this study, the indirect effect was verified using bootstrap to help in the understanding of the complex relationship of the proposed research structure. The indirect effect analysis showed that the perceived risk of PM has a statistically significant indirect effect on customer return on investment (β PM—trust and desire—customer return on investment = −0.736, *p* < 0.01) and the intention to travel to a tourism destination (β PM—trust and desire—customer return on investment—willingness to visit a tourism destination = −0.432, *p* < 0.01). Furthermore, both trust in the tourism destination (β Trust—customer return on investment—willingness to visit a tourism destination = 0.115, *p* < 0.01) and desire for the tourism destination (β Desire—customer return on investment—willingness to visit a tourism destination = 0.455, *p* < 0.01) were found to have a statistically significant indirect effect on the intention to travel to a tourism destination. Therefore, the theoretical framework of this study successfully proves the mediating effect of trust in and desire for tourism destinations and customer return on investment. Details are shown in Table 2.

### 4.3. Structural Invariance Model Assessment

**Figure 2:**
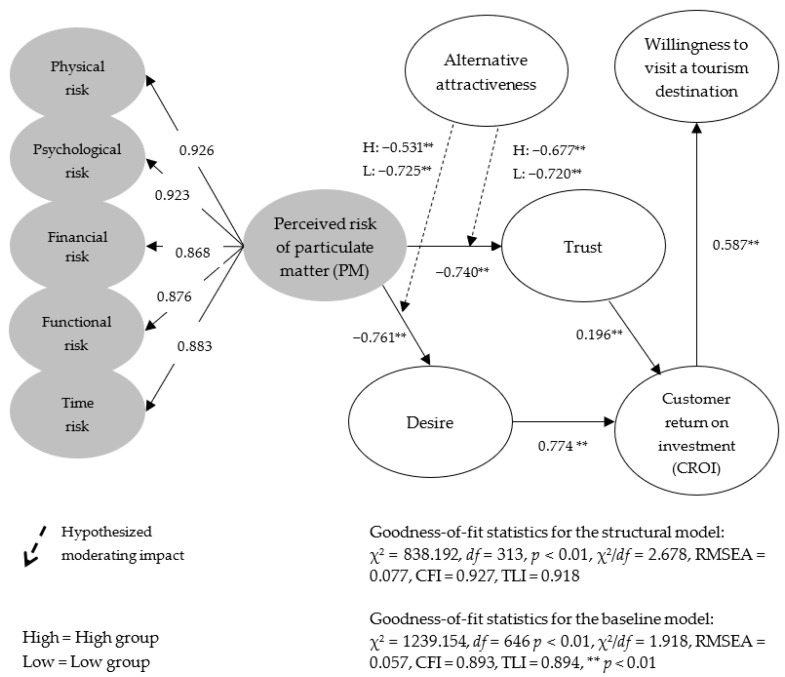
Structural model results and hypotheses testing.

This study performed an invariance test to verify the moderating effect of alternative attractiveness on the relationship between perceived risk for PMs and trust in and desire for tourism destinations. Hypotheses 6a and 6b were verified by classifying the 285 respondents into a high alternative attractiveness group (179 people) and a low alternative attractiveness group (106 people). The analysis showed that alternative attractiveness (Δχ^2^(1) = 7.004, *p* < 0.05) has a statistically significant moderating effect on the relationship between perceived risk of PM and trust in the tourism destination. However, alternative attractiveness (Δχ^2^(1) = 2.723, *p* > 0.05) was found to have no statistically significant moderating effect on the relationship between perceived risk of PM and desire for the tourism destination. Accordingly, Hypothesis 6a was supported, while Hypothesis 6b was not supported. These results reveal that there is a partially significant difference in each relationship presented in this study based on alternative attractiveness. This can be considered a very meaningful result. In addition, our research results agree with the findings of Jones et al. [34] and Nagengast et al. [45] claiming that consumer behavior can be either positive or negative depending on the alternatives’ attractiveness. Specific detailed results are shown in Table 3 and Figure 2.

## 5. Discussions

This study was designed to help understand the effect of perceived risk of PM on the trust in and desire for a tourism destination, customer return on investment, and willingness to visit a tourism destination. The perceived risk for PMs was divided into five categories, and the moderating role of alternative attractiveness on the relationship between perceived risk for PMs and trust in and desire for tourism destinations was verified. To achieve the purpose of this study, a quantitative methodology was used, and all measurement items confirmed through model validation were confirmed to have a statistically significant level of reliability and validity. Through the conceptual framework presented in this study, it was found that the perceived risk of PM satisfactorily explains the negative effect on trust in and desire for a tourism destination, and successfully describes the process of forming customer return on investment and willingness to travel to a tourism destination. Moreover, the perceived risk of PM was satisfactorily reflected in the higher-order factor framework, and the important mediating effects of trust in and desire for a tourism destination and customer return on investment were successfully revealed. In addition, a partial moderating role of alternative attractiveness was confirmed by verifying the moderating effect. Accordingly, the results of this study confirmed that the perceived risk of PM has a serious negative impact on the tourism industry in Korea, and highlighted the need to explore and actively manage the negative effects of PM on tourism destinations in the future. 

The analysis showed that the risk of PM perceived by consumers has a very negative effect on trust in and desire for tourism destinations. These results suggest that occurrence of PM can negatively affect various groups. Specifically, it is highly likely to negatively affect tourists (e.g., loss of tourism opportunities in the area and an increase in various types of risk during the tour) and the local community at the tourism destination (e.g., a reduction in tourism income and the number of jobs for local residents). Similar to this study, many previous studies have mentioned that the risk perceived by consumers has a negative effect on consumers’ decision-making and behavioral intention [11,19,20,21]. Hence, considering the results of previous studies and this study comprehensively, the risk of PM perceived by consumers is very likely to have a serious negative impact on tourism destinations. Next, the trust in and desire for the tourism destination had a positive effect on customer return on investment. As mentioned in many studies, such a result indicates the positive effect of the trust and desire perceived by tourists [10,26]. The results of this study are different from those of previous studies that presented the positive effects of trust and desire. Specifically, this study has great significance in that it demonstrates the positive effect of trust and desire from a financial point of view, unlike previous studies. Lastly, customer return on investment was found to have a positive effect on the intention to visit the tourism destination. Such a result is meaningful, as it proves the importance of financial effectiveness among various intentions to visit the tourism destination. Hence, considering the results of this study, there is a need to manage the perceived risk of PM actively in order to improve trust in and desire for tourism destinations, and form the intention to visit the tourism destination willingly.

In this study, alternative attractiveness had a significant moderating effect on the relationship between perceived risk of PM and trust in and desire for the tourism destination. Such a result implies that when there is a threat factor perceived by tourists, trust in and desire for a tourism destination decreases, and further, if alternative attractiveness is high, tourists are more likely to change their tourism destination or not revisit it. Previous studies have presented arguments about how alternatives are considered by consumers. If a customer feels the alternative is insufficiently attractive due to its moderating effect, they may become favorably inclined toward the company. On the other hand, if the customer determines that the alternative is powerfully attractive, they may develop a strong intention and behavior to switch to another company [34,44,45]. Accordingly, various measures to protect tourists from PM should be prepared, even if a tourism destination is directly affected by PM. For example, many air purifiers should be installed in the internal facilities of tourism destinations, and the best cleaning conditions should be maintained to minimize contamination of the internal air. Moreover, masks can be prepared in preparation for using external facilities, and customers should be notified of the PM concentration and air quality. Moreover, various landscaping facilities can be installed at tourism destinations to minimize the concentration of PM. Through this method, practitioners of tourism destinations need to present various methods and preventive measures to protect customers from PM.

## 6. Conclusions

The risk of PM perceived by tourists is a very important issue at this point wherein the frequency of PM occurrence is continuously increasing. In addition, investigating the effect of the perceived risk of PM on the emotional response and behavioral intention of tourists toward tourism destinations, and verifying the moderating role of alternative attractiveness in the relationship between perceived risk of PM and emotional response of tourists can be considered very important. However, the studies that classified the perceived risk of PM into five sub-factors and examined the consumers’ emotional reactions and behavioral intentions toward tourism destinations are very limited. Accordingly, in this study, the effect of the perceived risk of PM on the emotional response and behavioral intention of tourists was investigated, and the moderating role of alternative attractiveness was examined. As shown by the results of this study, perceived risk of PM had a serious negative impact on the emotional responses and behavioral intentions of tourists. In addition, a significant mediating effect of trust in and desire for tourism destinations and customer return on investment was verified within the theoretical framework of this study, and a partial moderating effect of alternative attraction was also found. Hence, the findings of this study can be said to have provided an important insight to researchers and practitioners examining the tourism industry.

This study presented various meaningful findings, but it still has some limitations. First, it has a limitation in generalizing it to other countries and places with different geographical environments and cultures since the survey of this study was conducted in Korea. Second, there is a partial limitation in understanding the severity of PM since PM does not occur in all countries and regions, and there is a limitation in applying the results of this study to all countries and regions. Third, the differences in the risk of PM perceived by tourists according to demographic characteristics were not distinguished. Hence, there is a need to expand the study by comparing countries and regions where PM occurs or does not occur in future studies. Further, there is a need to extend the perception risk of PM to studies that can verify differences according to demographic characteristics. Lastly, although our sample size (n = 285) is greater than the minimum number of samples size (n = 110), increasing the sample size can better demonstrate the effectiveness of the hypothesized theoretical framework. For future research, utilizing the greater number of samples are therefore recommended. 

## Figures and Tables

**Table 1:** Measurement model results and correlations.

	(1)	(2)	(3)	(4)	(5)	(6)	(7)	(8)	(9)	(10)
(1) PHY(R)	1.000									
(2) PSY(R)	0.633 ^a^ (0.400) ^b^	1.000								
(3) FIN(R)	0.537 (0.288)	0.628 (0.394)	1.000							
(4) FUN(R)	0.598 (0.357)	0.634 (0.401)	0.672 (0.451)	1.000						
(5) TI(R)	0.435 (0.189)	0.654 (0.427)	0.413 (0.170)	0.508 (0.258)	1.000					
(6) TR	−0.511 (0.261)	−0.673 (0.452)	−0.408 (0.166)	−0.616 (0.379)	−0.619 (0.383)	1.000				
(7) DR	−0.614 (0.376)	−0.674 (0.454)	−0.568 (0.322)	−0.606 (0.367)	−0.662 (0.438)	0.639 (0.408)	1.000			
(7) CRI	−0.682 (0.465)	−0.391 (0.152)	−0.643 (0.413)	−0.674 (0.454)	−0.686 (0.470)	0.710 (0.504)	0.679 (0.459)	1.000		
(7) WV	−0.535 (0.286)	−0.373 (0.139)	−0.690 (0.476)	−0.654 (0.427)	−0.586 (0.343)	0.570 (0.324)	0.573 (0.328)	0.566 (0.320)	1.000	
(7) AA	−0.591 (0.345)	−0.576 (0.331)	−0.677 (0.458)	−0.658 (0.332)	−0.318 (0.101)	0.494 (0.244)	0.469 (0.219)	0.574 (0.329)	0.597 (0.356)	1.000
Mean	5.131	5.016	4.993	4.644	4.786	2.734	3.014	2.705	2.883	4.621
SD	1.213	1.388	1.215	1.495	1.314	1.098	1.262	1.302	1.339	1.535
CR	0.827	0.819	0.828	0.813	0.829	0.884	0.857	0.885	0.858	0.844
AVE	0.614	0.601	0.616	0.596	0.620	0.718	0.666	0.719	0.669	0.644

Note. PHY(R): physical risk, PSY(R): psychological risk, FIN(R): financial risk, FUN(R): functional risk, TI(R): time risk, TR: trust, DR: desire, CRI: customer return investment, WV: willingness to visit a tourism destination, AA: alternative attractiveness. Goodness-of-fit statistics for the structural model: χ^2^ = 830.677, df = 360, *p* < 0.001, χ^2^/df = 2.307, RMSEA = 0.068, CFI = 0.941, TLI = 0.928 ^a^ Correlations, ^b^ Squared correlations.

**Table 2:** Structure model results.

Hypothesized Paths			Coefficients	*t*-Values
H1: PR	→	TR	−0.740	−11.608 **
H2: PR	→	DR	−0.761	−12.033 **
H3: TR	→	CRI	0.196	4.383 **
H4: DR	→	CRI	0.774	14.147 **
H5: CRI	→	IV	0.587	10.175 **
Indirect effect: β _PR → TR & DR → CRI_ = −0.736 ** β _PR → TR & DR → CRI → WIL_ = −0.432 ** β _TR → CRI → WIL_ = 0.115 ** β _DR → CRI → WIL_ = 0.455 **	Explained variance: R^2^ PHY(R) = 0.767 R^2^ PSY(R) = 0.753 R^2^ FIN(R) = 0.851 R^2^ FUN(R) = 0.858 R^2^ TI(R) = 0.779			R^2^ TR = 0.550 R^2^ DR = 0.582 R^2^ CRI = 0.810 R^2^ WIL = 0.344

** *p* < 0.01. Note. PR: perceived risk, PHY(R): physical risk, PSY(R): psychological risk, FIN(R): financial risk, FUN(R): functional risk, TI(R): time risk, TR: trust, DR: desire, CRI: customer return investment, WV: willingness to visit a tourism destination, AA: alternative attractiveness Goodness-of-fit statistics for the structural model: χ^2^ = 838.192, df = 192, *p* < 0.001, χ^2^/df = 2.678, RMSEA = 0.077, CFI = 0.927, TLI = 0.918.

**Table 3:** Results of invariance test for structural models.

Paths	High AA Group (*n* = 176)	Low AA Group (*n* = 106)	Baseline Model (Freely Estimated)	Nested Model (Constrained to Be Equal)
	Β	β		
H6a: PR → TR	−0.531 **	−0.725 **	χ^2^ (646) = 1239.154	χ^2^ (647) = 1246.158 ^a^
H6b: PR → DR	−0.677 **	−0.720 **	χ^2^ (646) = 1239.154	χ^2^ (647) = 1241.877 ^b^
Chi-square test: ^a^ Δχ^2^ (1) = 7.004, *p* < 0.05 ^b^ Δχ^2^ (1) = 2.723, *p* > 0.05		Hypotheses testing: H6a: Supported H6b: Not supported		Goodness-of-fit statistics for the baseline model: χ^2^ = 1239.154, *df* = 646 *p* < 0.01, χ^2^/*df* = 1.918, RMSEA = 0.057, CFI = 0.893, TLI = 0.894 * *p* < 0.05, ** *p* < 0.01

Note. PR: perceived risk, TR: trust, DR: desire, AA: alternative attractiveness.

## Data Availability

The data used in this research are available upon request from the corresponding author. The data are not publicly available due to restrictions i.e., privacy or ethical.
